# Are physical performance and frailty assessments useful in targeting and improving access to adjuvant therapy in patients undergoing resection for pancreatic cancer?

**DOI:** 10.1007/s00423-023-02828-1

**Published:** 2023-02-14

**Authors:** S. Powell-Brett, J. Hodson, R. Pande, S. Mann, Alice Freer, Zoe Wyrko, Clare Hughes, J. Isaac, R. P. Sutcliffe, K. Roberts

**Affiliations:** 1 Department of Hepatopancreatobiliary Surgery and Liver Transplantation, Queen Elizabeth Hospital, Birmingham, B15 2TH Birmingham, UK; 2 Research Development and Innovation, Queen Elizabeth Hospital Birmingham, Birmingham, UK; 3 Department of Geriatric Medicine, Queen Elizabeth Hospital Birmingham, Birmingham, UK; 4 Institute of Immunology and Immunotherapy, University of Birmingham, Birmingham, UK

**Keywords:** Pancreatic cancer, Adjuvant chemotherapy, Pancreatic exocrine insufficiency, Nutrition, Frailty, Prehabilitation

## Abstract

**Background:**

Many patients fail to receive adjuvant chemotherapy following pancreatic cancer surgery. This study implemented a multimodal, multidisciplinary approach to improving recovery after pancreatoduodenectomy (the ‘Fast Recovery’ programme) and measured its impact on adjuvant chemotherapy uptake and nutritional decline. The predictive accuracies of a bundle of frailty and physical performance assessments, with respect to the recipient of adjuvant chemotherapy, were also evaluated.

**Results:**

The *N* = 44 patients treated after the introduction of the ‘Fast Recovery’ programme were not found to have a significantly higher adjuvant chemotherapy uptake than the *N* = 409 treated before the pathway change (80.5 vs. 74.3%, *p* = 0.452), but did have a significantly lower average weight loss at six weeks post-operatively (mean: 4.3 vs. 6.9 kg, *p* = 0.013). Of the pre-operative frailty and physical performance assessments tested, the 6-min walk test was found to be the strongest predictor of the receipt of adjuvant chemotherapy (area under the ROC curve: 0.91, *p* = 0.001); all patients achieving distances ≥ 360 m went on to receive adjuvant chemotherapy, compared to 33% of those walking < 360 m.

**Conclusions:**

The multimodal ‘Fast Recovery’ programme was not found to significantly improve access to adjuvant chemotherapy, but did appear to have benefits in reducing nutritional decline. Pre-operative assessments were found to be useful in identifying patients at risk of non-receipt of adjuvant therapies, with markers of physical performance appearing to be the best predictors. As such, these markers could be useful in targeting pre- and post-habilitation measures, such as physiotherapy and improved dietetic support.

**Supplementary Information:**

The online version contains supplementary material available at 10.1007/s00423-023-02828-1.

## Introduction

Surgical resection is the cornerstone of management of ‘resectable’ pancreatic cancer but, on its own, is associated with less than a 10% chance of cure [1]. Evolving regimens of adjuvant chemotherapy have markedly improved patient survival [1–4]. Despite the fundamental role of adjuvant chemotherapy, a significant proportion of patients do not receive it [5–8]. A critical review of the literature reveals that elderly and/or frail patients are most at risk of undertreatment [9, 10]. There is also widespread variation in the use of chemotherapy within healthcare systems, suggesting that the attitude and practice of individual teams is a major contributor to uptake rates.

It is recognised that baseline functional status influences both access to and outcomes following surgery and chemotherapy [5, 10–12]. There is also an increasing body of evidence supporting the use of prehabilitation to improve access to treatment, outcomes, and quality of life [13–16]. These benefits are shown even in the few studies looking at prehabilitation specifically for patients undergoing resection for pancreatic cancer [ 16–19]. However, especially in the setting of resectable pancreatic cancer, it is difficult to identify those in most need of optimisation and in a resource-limited environment, and a ‘one-size fits all approach’ may not be appropriate. Therefore, being able to identify patients at risk would be invaluable in allocating pre- and post-operative resources, such as targeted physiotherapy, dietetic support, and geriatrician input.

Patients with pancreatic cancer are frequently malnourished at diagnosis, with poor cardio-pulmonary reserve and sarcopenia. This poor baseline function increases the risk of post-operative complications and reduces the likelihood of receiving adjuvant chemotherapy [10, 20, 21]. Pancreatic exocrine insufficiency (PEI) is common following pancreatic resection and contributes to reduced survival; despite this, there is widespread undertreatment of PEI [ 22–24 ]. Several other factors can contribute to malnutrition in this patient cohort such as bile salt malabsorption, small intestine bacterial overgrowth and type 3c diabetes [25]. Identifying those at risk of not receiving adjuvant therapy, addressing frailty and nutritional failure and optimising recovery from surgery may increase the number of patients receiving and completing adjuvant chemotherapy.

With the understanding that there are multiple factors contributing to the underuse of chemotherapy, a multimodal, multi-therapy pathway change (the ‘Fast Recovery’ programme) was developed. This was designed to identify those at risk of non-receipt of adjuvant chemotherapy, deliver targeted physiotherapy and ensure adequate pancreatic enzyme replacement therapy (PERT) in all patients (as per the U.K. NICE (National Institute for Clinical Excellence) guidelines). The primary aims of this study were to assess whether the ‘Fast Recovery’ pathway could improve the uptake of adjuvant chemotherapy and be effective in preventing nutritional decline, as quantified by post-operative weight loss. The study also aimed to assess which, if any, of the bundle of frailty, cognitive, nutritional and physical functioning assessments implemented as part of the programme were most applicable to this group and were useful in identifying patients at risk of not receiving adjuvant therapy.

## Methods

This was a prospective, single-centre, observational cohort study before and after implementation of the ‘Fast Recovery’ programme at University Hospitals Birmingham (UHB), a specialist centre for pancreatic resection.

### Pathway prior to the ‘Fast Recovery’ programme

Prior to the pathway change, patients were seen in the clinic once pre-operatively; physiotherapy was only given for those considered ‘high risk’. After discharge, patients had a single surgical follow-up at 4–6 weeks before being referred back to their local oncology department for consideration of adjuvant chemotherapy.

### Development and implementation of the ‘Fast Recovery’ programme

**Fig. 1:**
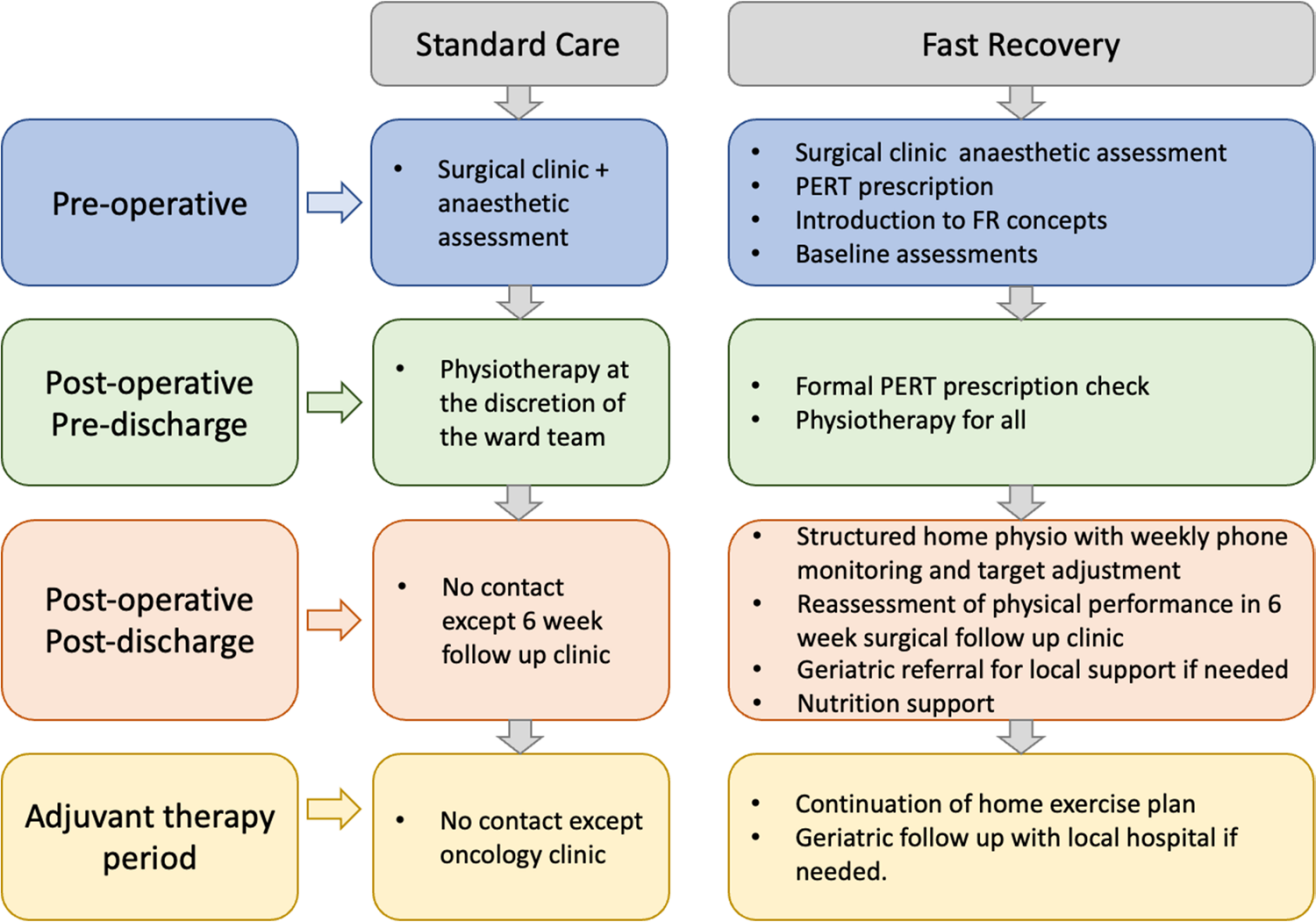
Structure of the standard pathway and the novel ‘Fast Recovery’ pathway

Figure 1 gives an overview of the ‘Fast Recovery’ pathway (compared to the standard pathway). A frailty assessment bundle, developed in conjunction with geriatricians, was implemented for pre-operative assessment. A range of assessments was included in the bundle, with five frailty and activities of daily living (ADL) assessments (Katz IADL, Lawton ADL, Fried scale score, Clinical Frailty scale and the Edmonton Frail scale), [ 26–30] one cognitive assessment (the Montreal cognitive assessment [MoCA]), [31] three assessments of physical functioning (the short physical performance battery, the six-minute walk test and hand grip strength) [32–34] and a nutritional assessment (the mini nutritional assessment) [35].

At the pre-operative contact, patients underwent the ‘Fast Recovery’ assessment bundle, had the importance of activity and exercise emphasised to them and received a prescription for PERT, in line with the planned pathway changes. PERT was prescribed in a standardised fashion, alongside practical advice and a daily proton pump inhibitor [24]. The next pathway interaction was in the post-operative period, prior to discharge. At this time, the physical functioning assessments were repeated, and all patients received daily physiotherapy input to introduce an exercise programme. This included graded step count goals and High Intensity Interval Training (HIIT) for patients to complete during their post-operative recovery. On discharge, patients remained in contact with the physiotherapy team to enable continued, graded HIIT therapy and walking plans.

**Fig. 2:**
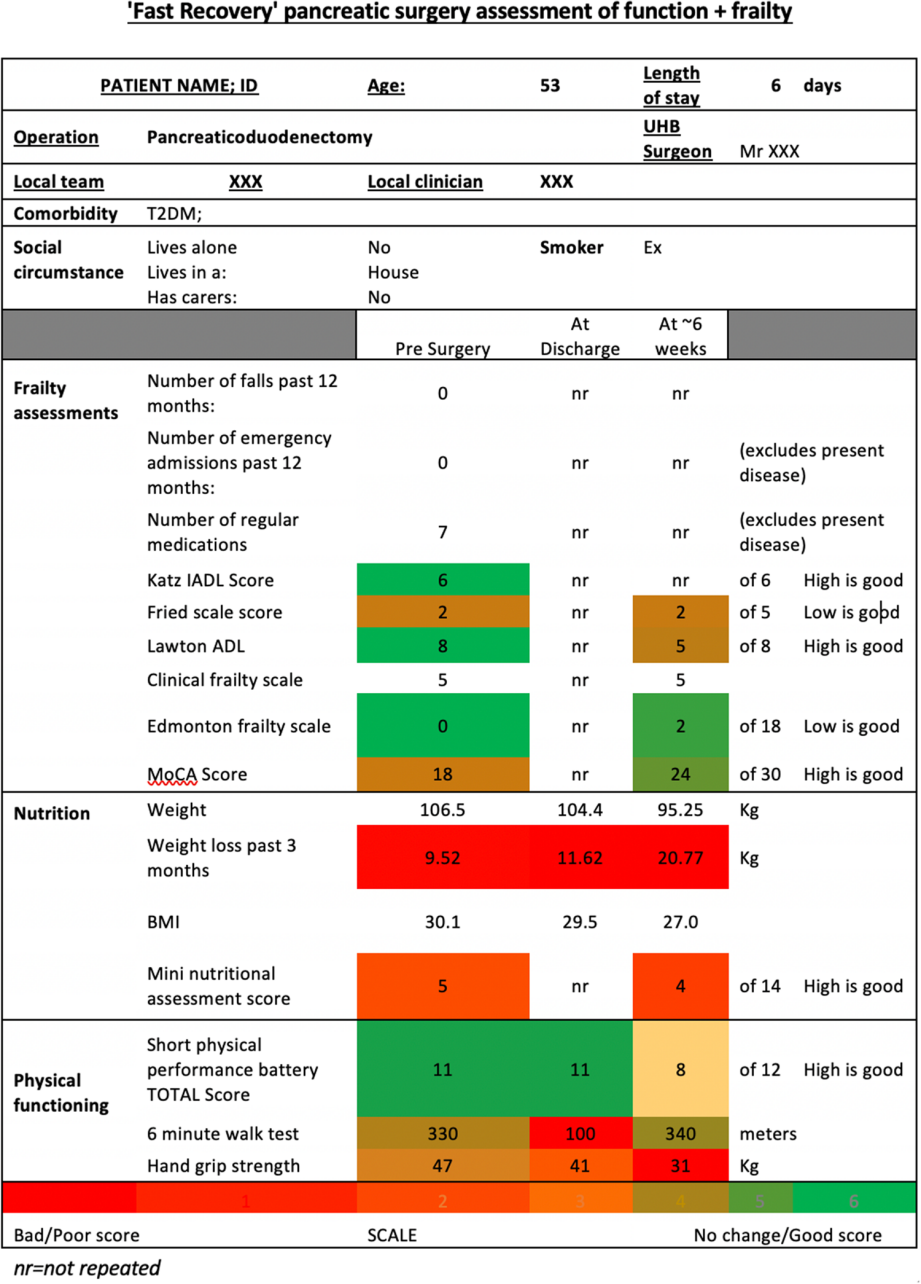
Summary sheet of assessment results

Patients returned at six weeks post-operatively for routine outpatient clinical review. At this visit, the full assessment bundle was repeated, and physiotherapy input and nutritional advice were given, as required. Data from all assessments were compiled into a summary document (Fig. 2), which was forwarded selectively to local geriatricians. Those deemed to be frail could then have their local support optimised to aid recovery.

Funding was obtained for 12 months of physiotherapy support from the PCUK Clinical Pioneers Scheme. The study began in April 2018 and was completed in April 2019.

### Study cohort

Patients undergoing pancreatoduodenectomy between January 2007 and March 2019 were included. The ‘Fast Recovery’ cohort consisted of patients operated on between April 2018 and March 2019; data for these patients were collected prospectively. The historical cohort consisted of patients operated prior to April 2018, who were retrospectively identified from a departmental database. As a diagnosis of pancreatic cancer is often proven only after surgery, the pathway changes were implemented in all patients undergoing surgery for suspected pancreatic cancer; however, only those with a histological diagnosis of pancreatic cancer were included in the final analysis. Patients undergoing neoadjuvant therapy, distal pancreatectomy or total pancreatectomy were excluded to keep a homogenous cohort regarding chemotherapy needs.

### Statistical methods

Nominal variables were compared between the cohorts using Fisher’s exact tests or Chi^2^ tests for factors with two or more than two levels, respectively, whilst ordinal and continuous variables were assessed using Mann–Whitney *U* tests. For analysis of the changes in patient weight, within-group comparisons were performed using paired *t*-tests, with independent samples *t*-tests used to compare between groups at each time point. For the ‘Fast Recovery’ cohort, the predictive accuracy of the assessment bundle with respect to the receipt of adjuvant chemotherapy was quantified using the area under the receiver operating characteristic curve (AUROC). Comparisons of the assessment bundle between the pre-operative assessment and subsequent assessments were performed using Wilcoxon’s signed-rank tests.

Continuous data are reported as mean ± standard deviation where normally distributed or as median (interquartile range; IQR) otherwise. Patients with missing data are excluded from the analysis of the affected variable. All analyses were performed using IBM SPSS 22 (IBM Corp. Armonk, NY), with *p* < 0.05 deemed to be indicative of statistical significance throughout.

## Results

### Demographics

Data were available for a total of *N* = 453 patients, of whom *N* = 44 (9.7%) were treated during the ‘Fast Recovery’ era. Comparisons of demographic and surgical outcomes between the two eras are reported in Table 1. Patient demographics were similar in the two eras, with no significant differences detected in the distributions of age, gender or ethnicity.

**Table 1:** Comparison of demographics and surgical outcomes between eras

		Fast Recovery		
	*N*	*No*	*Yes*	*p***-**value
Referring centre	435			0.443
* University Hospital Birmingham*		108 (27.6%)	8 (18.2%)	
* Centre A*		51 (13.0%)	10 (22.7%)	
* Centre B*		54 (13.8%)	4 (9.1%)	
* Centre C*		36 (9.2%)	6 (13.6%)	
* Centre D*		36 (9.2%)	3 (6.8%)	
* Centre E*		19 (4.9%)	4 (9.1%)	
* Centre F*		17 (4.3%)	3 (6.8%)	
* Centre G*		16 (4.1%)	2 (4.5%)	
* Centre H*		11 (2.8%)	0 (0.0%)	
*Other centres*		43 (11.0%)	4 (9.1%)	
Age at surgery (years)	453	68.4 (61.5–73.3)	69.6 (64.0–74.9)	0.345
Gender (% male)	453	217 (53.1%)	29 (65.9%)	0.113
Ethnicity (% white)	453	390 (95.4%)	39 (88.6%)	0.072
BMI (kg/m^2^)	442	24.9 (22.3–28.3)	26.4 (24.2–27.8)	0.083
Current smoker	453	52 (12.7%)	4 (9.1%)	0.633
Charlson comorbidity index				0.317*
* 2–4*		178 (43.5%)	20 (45.5%)	
* 5*		116 (28.4%)	17 (38.6%)	
* 6* +		115 (28.1%)	7 (15.9%)	
Overall tumour stage	447			0.263*
* 1*		61 (15.1%)	7 (16.3%)	
* 2*		141 (34.9%)	19 (44.2%)	
* 3*		202 (50.0%)	17 (39.5%)	
T-stage	447			0.984*
* T1*		68 (16.8%)	7 (16.3%)	
* T2*		306 (75.7%)	33 (76.7%)	
* T3*		30 (7.4%)	3 (7.0%)	
N-stage	453			0.328*
* N0*		67 (16.4%)	8 (18.2%)	
* N1*		139 (34.0%)	18 (40.9%)	
* N2*		203 (49.6%)	18 (40.9%)	
CA19-9 (U/ml)	378	169 (44–687)	369 (108–985)	**0.047**
Pre-operative jaundice	453	354 (86.6%)	40 (90.9%)	0.636
Pre-operative biliary drainage	452	260 (63.7%)	7 (15.9%)	** < 0.001**
PERT	429	242 (62.9%)	40 (90.9%)	** < 0.001**
R-status	453			0.865
* R0*		283 (69.2%)	30 (68.2%)	
* R1*		126 (30.8%)	14 (31.8%)	
90-day mortality	453	25 (6.1%)	3 (6.8%)	0.745
Length of stay (days)**	441	9 (7–13)	9 (7–14)	0.593

Data are reported as *N* (%), with *p*-values from Chi^2^/Fisher’s exact test, or as median (interquartile range), with *p*-values from Mann–Whitney *U* tests, unless stated otherwise. Bold *p*-values are significant at *p* < 0.05

^*^p-value from Mann–Whitney U test, as the factor is ordinal

^**^Excluding post-operative in-hospital deaths

*BMI*, body mass index; *PERT*, pancreatic enzyme replacement therapy

### Adjuvant chemotherapy

For analysis of adjuvant chemotherapy, those patients who died with 90 days post-operatively were excluded (*N* = 28, 6.2%), as they did not have the potential to receive it, as were *N* = 7 (1.5%) for whom the adjuvant chemotherapy status was not recorded. Of the remainder, 80.5% of patients in the ‘Fast Recovery’ era received adjuvant chemotherapy, which was not a significant improvement over the 74.3% in the prior era (*p* = 0.452, Table 2). However, this was equivalent to a relative risk reduction in the likelihood of not receiving adjuvant chemotherapy of 24.2% (95% CI − 42.6%, 64.5%); hence, the lack of significance may, in part, reflect insufficient statistical power. In those that did not commence chemotherapy, the most common reasons were due to being frail/unfit (30.3%), post-operative complications (19.2%), early recurrence (17.2%), and patient choice (17.2%); the distribution of these reasons was found to be similar in the two eras (*p* = 0.866). For those with early recurrence, all (*N* = 2) were switched to palliative chemotherapy in the ‘Fast Recovery’ cohort compared to only 66.7% (16/24) in the historical era, with the remainder receiving no further treatment. The chemotherapeutic agent used differed significantly between eras (*p* < 0.001), with gemcitabine and capecitabine being the predominant approach in the ‘Fast Recovery’ era (51.5%), whilst gemcitabine alone was the most common regime in the earlier era (73.4%). In those that received adjuvant chemotherapy, there was a tendency for the ‘Fast Recovery’ era to have a higher likelihood of completing six or more cycles, although this did not reach statistical significance (81.8% vs. 66.8%, *p* = 0.108).

**Table 2:** Comparison of adjuvant chemotherapy rates between eras

		Fast Recovery		
	*N*	*No*	*Yes*	*p*-value
Adjuvant chemotherapy	418	280 (74.3%)	33 (80.5%)	0.452
Reason for no chemotherapy*	99			0.866
* Frail/unfit*		29 (31.9%)	1 (12.5%)	
* Post-operative complication*		17 (18.7%)	2 (25.0%)	
* Early recurrence*		15 (16.5%)	2 (25.0%)	
* Patient choice*		15 (16.5%)	2 (25.0%)	
* No referral*		13 (14.3%)	1 (12.5%)	
* Oncology decision*		2 (2.2%)	0 (0.0%)	
Chemotherapy type**	304			** < 0.001**
* Gemcitabine*		199 (73.4%)	4 (12.1%)	
* Gemcitabine/capecitabine*		55 (20.3%)	17 (51.5%)	
* Folfirinox*		16 (5.9%)	11 (33.3%)	
* Capecitabine*		1 (0.4%)	1 (3.0%)	
*N* cycles (6 +)**	271	159 (66.8%)	27 (81.8%)	0.108
Chemo complete**	275	158 (65.3%)	23 (69.7%)	0.698
Reason for incomplete chemo***	84			0.868
* Toxicity/side effects*		39 (52.7%)	7 (70.0%)	
Early recurrence****		24 (32.4%)	2 (20.0%)	
* Frail/unfit*		5 (6.8%)	1 (10.0%)	
* Other illness*		3 (4.1%)	0 (0.0%)	
* Death*		2 (2.7%)	0 (0.0%)	
* Patient choice*		1 (1.4%)	0 (0.0%)	

Patients that died within 90 days of surgery (*N* = 28) were excluded from all analyses. Data are reported as *N* (%), with *p*-values from Chi^2^/Fisher’s exact tests. Bold *p*-values are significant at *p* < 0.05

^*^In patients not receiving chemotherapy

^**^In patients receiving chemotherapy

^***^In patients not completing chemotherapy

^****^ Further treatment after recurrence is discussed in the text

Patients treated in the ‘Fast Recovery’ era had a significantly lower rate of pre-operative biliary drainage (15.9% vs. 63.7%, *p* < 0.001) and were also found to have significantly higher CA19-9 (median: 369 vs. 169 U/ml, *p* = 0.047) than those treated prior to this era. Subgroup analysis within those presenting with jaundice found the difference in CA19-9 to be most pronounced in those that were not treated with pre-operative biliary drainage (median: 443 vs. 200 U/ml, *p* = 0.013); no significant difference between eras was detected for jaundiced patients treated with biliary drainage (median: 191 vs. 173 U/ml, *p* = 0.861, Supplementary Table 1).

Surgical outcomes were similar in the two eras, with no significant differences in the *R*-status (*p* = 0.865), 90-day post-operative mortality (6.8% vs. 6.1%, *p* = 0.745) or average lengths of stay (median: 9 vs. 9 days, *p* = 0.593). Complication rates were also similar in the two eras (Supplementary Table 2).

### Post-operative weight loss and PERT prescribing

PERT use increased significantly after the implementation of the ‘Fast Recovery’ pathway, from 62.9 to 90.9% (*p* < 0.001). Of the four patients on the ‘Fast Recovery’ pathway that did not receive PERT, one died in the immediate post-operative period, two declined, and no reason was given for the final patient. Patient weights were not routinely recorded at follow-up until 2011; hence, patients with surgery prior to this were excluded from the analysis of changes in weight. Prior to surgery, the average weights were similar in the two eras (*p* = 0.229, Table 3). In the ‘Fast Recovery’ era, no significant change in weight was observed after surgery (mean difference: + 0.5 kg, *p* = 0.486), whilst significant weight loss was observed in patients from the earlier era (− 1.7 kg, *p* < 0.001). At the six-week review, weight data were recorded for 65.9% (*N* = 29) of patients from the ‘Fast Recovery’ era, and 56.1% (*N* = 165) of the earlier era. Significant weight loss was observed in both groups, relative to pre-operative measurements (both *p* < 0.001). However, the degree of weight loss at six weeks was significantly smaller for patients in the ‘Fast Recovery’ era, with a mean reduction of 4.3 vs. 6.9 kg (*p* = 0.013).

**Table 3:** Change in patient weight

	Fast Recovery				
	No		Yes		
Weight (Kg)	*N*	*Mean* ± *SD*	*N*	*Mean* ± *SD*	*p*-value**
Pre-operative	294	73.1 ± 15.6	44	76.1 ± 14.4	0.229
Pre-discharge	273	71.6 ± 15.6	43	76.2 ± 13.6	0.069
* Change vs. pre-operative*	273	− 1.7 ± 4.1	43	0.5 ± 4.2	**0.001**
p-value*	**-**	***p*** < 0.001*	**-**	*p *= 0.486*	**-**
Six-week review	165	66.7 ± 14.8	29	72.6 ± 12.8	**0.047**
* Change vs. pre-operative*	165	− 6.9 ± 5.1	29	− 4.3 ± 5.7	**0.013**
* p*-value*	-	*p* < 0.001*	-	*p* < 0.001*	-

Bold *p*-values are significant at *p* < 0.05

^*^p-value from a paired *t*-test, assessing the change in weight for the two groups separately; only those with weight measurements at both time points were included in this analysis

^**^p-value from an independent samples *t*-test, comparing between the two groups

### Changes over time in the assessment bundle

The bundle of frailty, physical performance, cognitive and nutritional assessments were then further assessed for the patients treated as part of the ‘Fast Recovery’ programme. Of the *N* = 44 patients treated in the ‘Fast Recovery’ era, *N* = 40 attended the pre-operative assessment. The remaining *N* = 4 were missed owing to appointment rescheduling; they still received the pathway interventions, but subsequent assessments were not performed, owing to the lack of baseline data. For the *N* = 40 attending the pre-operative assessment, the assessment bundle was performed for *N* = 36 (*N* = 4 were unable to undergo assessment due to lack of space or time). Post-operative assessments were performed in *N* = 34 patients (*N* = 1 died before discharge, *N* = 1 declined, *N* = 2 were too unwell, and *N* = 2 were missed due to lack of assessor availability). A total of *N* = 28 patients returned for the six-week follow-up and were reassessed (*N* = 2 patients had died before this, *N* = 3 declined to engage, *N* = 3 were missed due to lack of assessor availability, *N* = 3 patients were seen at their local hospital rather than UHB, and *N* = 1 was lost to follow-up).

There were low rates of pre-operative frailty, with 100% (33/33) of patients scoring maximum points for the Katz IADL (‘patient independent’), 86% (31/36) scoring maximum points for the Lawton ADL (‘high function’), 85% (29/34) scoring less than three points on the Edmonton Frail scale (‘not frail’), 83% (30/36) scoring less than three points on the Fried score (‘not frail’) and 83% (30/36) scoring less than four points on the Clinical Frailty Scale (‘not frail’). Comparisons of the bundle between the pre-operative and six-week follow-up assessments found only the Clinical Frailty Scale score to differ between assessments, with the mean score increasing from 2.4 to 2.9 (*p* = 0.030, Table 4). Considering that the frailty scores identified few patients as being frail and did not appear to change post-operatively, it was concluded that these assessments were unlikely to be useful in identifying frailty amongst this cohort.

**Table 4:** Analysis of assessment scores by measurement time in the Fast Recovery era

Assessment	Scale	Good score		Time point		*p*-value
			*N*	Pre-operative	Six-week review	
Katz IADL (% scoring 6*)	0–6	High	33	33 (100%)	NA**	NA**
Fried scale score	0–5	Low	10	0 (0–2)	2 (0–3)	0.289
Lawton ADL (% scoring 8*)	0–8	High	26	22 (85%)	24 (92%)	0.844
Clinical frailty scale	1–9	Low	26	2.4 ± 1.2	2.9 ± 1.0	**0.030**
Edmonton frail scale	0–18	Low	10	0 (0–2)	2 (1–2)	0.395
Mini nutritional assessment	0–14	High	10	11 (7–12)	9 (7–11)	0.180
MoCA score	0–30	High	18	26 (23–27)	26 (25–28)	0.100
Hand grip strength	Kg	High	20	31.9 ± 10.8	30.0 ± 9.2	0.211
Short physical performance (% scoring 12*)	0–12	High	22	15 (68%)	11 (50%)	0.215
Six-minute walk test	Metres	High	20	414 ± 133	390 ± 141	1.000

Only those patients with data recorded for the assessment at both time points were included in the analysis. Data are reported as median (interquartile range) or as mean ± standard deviation, unless stated otherwise, with *p*-values from Wilcoxon’s signed ranks tests. Bold *p*-values are significant at *p* < 0.05

^*^The majority of the cohort scored the maximum number of points pre-operatively; hence, the proportion of cases with this score is reported to allow clearer comparison between time points; however, the *p*-values are based on the actual observed values

^**^Katz IADL was originally planned to be assessed at the six-week review. However, it was dropped from the review after the study commenced since it was felt to be unnecessarily cumbersome, and all patients had achieved the maximum score at the pre-operative assessment

Comparisons of the remainder of the assessments in the bundle between the pre-operative clinic and six-week review are reported in Table 4, and the subset of physical performance assessments that were additionally performed pre-discharge is reported in Table 5. Hand grip strength (*p* = 0.031), short physical performance (*p* < 0.001) and the six-minute walk test (*p* < 0.001) were all found to decline significantly between the pre-operative and pre-discharge assessments. However, by the six-week review, all three of these assessments had improved to the point that they were not significantly different from pre-operative levels.

**Table 5:** Analysis of assessment scores by measurement time in the Fast Recovery era

Assessment	Scale	Good score		Time point		*p***-**value
			*N*	Pre-operative	Pre-discharge	
Hand grip strength	Kg	High	26	32.3 ± 10.5	29.6 ± 11.7	**0.031**
Short physical performance (% scoring 12*)	0–12	High	26	20 (77%)	6 (23%)	** < 0.001**
Six-minute walk test	Metres	High	25	427 ± 122	213 ± 101	** < 0.001**

Only those patients with data recorded for the assessment at both time points were included in the analysis. Data are reported as median (interquartile range) or as mean ± standard deviation, unless stated otherwise, with *p*-values from Wilcoxon’s signed ranks tests. Bold *p*-values are significant at *p* < 0.05

^*^The majority of the cohort scored the maximum number of points pre-operatively; hence, the proportion of cases with this score is reported to allow clearer comparison between time points; however, the p-values are based on the actual observed values

### Associations between assessment bundle and adjuvant chemotherapy

Analysis of the predictive accuracy of the assessment bundle, with respect to identifying those patients who went on to receive adjuvant chemotherapy in the ‘Fast Recovery’ cohort, excluded those patients who were unable to undergo the pre-operative assessments or that died post-operatively. For the remaining *N* = 34 of the pre-operative assessments considered, neither the cognition (MoCA) nor nutrition (mini nutritional score) scores were found to be significantly predictive of the receipt of adjuvant chemotherapy (Table 6). In addition, none of the pre-operative frailty assessments were found to be significantly predictive of this outcome (Katz IADL, Fried Scale, Lawton ADL, Clinical Frailty Scale or Edmonton Frail Scale), with the Clinical Frailty Scale (AUROC: 0.71, *p* = 0.087) and Edmonton Frail Scale (AUROC: 0.73, *p* = 0.064) being closest to statistical significance. However, two pre-operative physical performance assessments were found to be significantly predictive of the receipt of adjuvant chemotherapy. The strongest of these was the six-minute walk test, which returned an AUROC of 0.91 (*p* = 0.001); all patients (19/19) achieving distances ≥ 360 m went on to receive adjuvant chemotherapy, compared to 33% (3/9) of those walking < 360 m. The other significant predictor was the short physical performance test (AUROC: 0.72, *p* = 0.043), with adjuvant chemotherapy rates of 83% (20/24) in those scoring the full 12 points, compared to 56% (5/9) in those with lower scores. When the physical performance scores were reassessed post-operatively, the significant predictive accuracy of the six-minute walk test persisted (AUROC: 0.79, *p* = 0.019), although the short physical performance test did not reach significance on this analysis (AUROC: 0.73, *p* = 0.060).

**Table 6:** Analysis of assessment scores by adjuvant chemotherapy treatment in the Fast Recovery era

			Adjuvant chemotherapy					
Assessment	Scale	Good score	Yes		No		*AUROC (SE)*	
			*N*	*Statistic*	*N*	*Statistic*		*p***-**value
*Pre-operative assessments*								
Katz IADL (% scoring 6*)	0–6	High	26	26 (100%)	5	5 (100%)	0.50 (0.14)	1.000
Fried scale score	0–5	Low	27	1 (0–2)	7	1 (0–2)	0.52 (0.12)	0.896
Lawton ADL (% scoring 8*)	0–8	High	27	24 (89%)	7	6 (86%)	0.51 (0.12)	1.000
Clinical frailty scale	1–9	Low	27	2.3 ± 1.1	7	3.0 ± 0.8	0.71 (0.10)	0.087
Edmonton frail scale	0–18	Low	26	0 (0–2)	6	1 (1–2)	0.73 (0.09)	0.064
Mini nutritional assessment	0–14	High	27	10 (9–12)	7	11 (7–11)	0.59 (0.12)	0.507
MoCA score	0–30	High	19	26 (24–27)	6	25 (20–26)	0.68 (0.12)	0.201
Hand grip strength	Kg	High	20	31.3 ± 10.0	6	29.8 ± 11.1	0.55 (0.14)	0.733
Short physical performance (% scoring 12*)	0–12	High	22	17 (77%)	6	2 (33%)	0.72 (0.13)	**0.043**
Six-minute walk test	Metres	High	22	454 ± 112	6	277 ± 48	0.91 (0.06)	**0.001**
*Post-operative assessments*								
Hand grip strength	Kg	High	25	28.2 ± 11.0	7	25.2 ± 8.2	0.60 (0.12)	0.453
Short physical performance	0–12	High	25	8 (6–12)	7	6 (5–7)	0.73 (0.11)	0.060
Six-minute walk test	Metres	High	23	243 ± 99	7	150 ± 124	0.79 (0.13)	**0.019**

Patients that died post-operatively were excluded from the analysis (*N* = 3). Data are reported as median (interquartile range) or as mean ± standard deviation, unless stated otherwise, with *p*-values from Mann–Whitney U tests. Bold *p*-values are significant at *p* < 0.05

^*^The majority of the cohort scored the maximum number of points; hence, the proportion of cases with this score is reported to allow clearer comparison between groups; however, the *p*-values are based on the actual observed values

*AUROC*, area under the receiver operating characteristic curve; *SE*, standard error

## Discussion

The aims of the study were to assess whether the ‘Fast Recovery’ pathway could improve the uptake of adjuvant chemotherapy and be effective in preventing nutritional decline and to determine which, if any, of the frailty assessments are most applicable to this patient group.

The pathway was not associated with a significant increase in adjuvant chemotherapy uptake, with rates of 80.5% in the ‘Fast Recovery’ era, compared to 74.3% in the prior era. Despite the lack of statistical significance, there was some indication of the potential for benefit. The observed change in uptake was equivalent to a relative risk reduction of non-receipt of adjuvant chemotherapy of 24.2%, which is a potentially clinically relevant effect. In addition, where patients received adjuvant chemotherapy, those in the ‘Fast Recovery’ era tended to be more likely to receive six or more cycles, although again, this did not reach statistical significance (81.8% vs. 66.8%, *p* = 0.108). The lack of statistical significance in these analyses is likely to reflect insufficient statistical power, as a result of the small sample size in the ‘Fast Recovery’ era (*N* = 44) and the fact that the rate of adjuvant chemotherapy uptake was already relatively high in comparison to national rates prior to the introduction of the programme [5]. Based on the observed rates, a post-hoc power calculation estimated that a sample size of almost *N* = 1500 patients would be required to achieve 80% power for the comparison of adjuvant chemotherapy uptake. As such, further research in this area is warranted, potentially with a larger multi-centre trial, in order to recruit a sufficient sample size.

The pathway was found to be associated with significantly lower post-operative weight loss, implying that it was effective in preventing nutritional decline. This may have been, in part, a result of the introduction of PERT prescribing for all at pre-operative contact, which was a key aspect of the ‘Fast Recovery’ programme, and lead to 90.9% of patients receiving PERT, up from 62.9% prior to the introduction of the pathway. This is important, given the high prevalence of PEI in this group and the fact that PERT improves symptoms, maintains weight and may improve receipt of chemotherapy and survival [22, 23, 36]. The ‘Fast Recovery’ bundle also included regular check in with a physiotherapist in the post-operative period and discussion of the ‘Fast Recovery’ concepts at pre-operative review, which may have additionally helped ameliorate weight loss. However, it is likely that PERT is the key factor preventing nutritional decline; therefore, further iterations of this pathway will include structured PERT prescribing.

The final aim of the study was to identify the most useful assessments for predicting non-receipt of adjuvant chemotherapy. This found the physical performance tests to be the best predictors of this outcome, particularly the six-minute walk test, which was found to be a significant predictor when performed both pre- and post-operatively; all patients who could walk 360 m or more went on to receive adjuvant chemotherapy, compared to 33% of those who managed less than 360 m. The short physical performance test was also a significant predictor, with adjuvant chemotherapy rates of 83% in those scoring the full 12 points, compared to 56% in those with lower scores. The frailty assessments performed more poorly, with none being significantly predictive of non-receipt of adjuvant chemotherapy, and only the Clinical Frailty Scale and Edmonton Frail Scale showing any potential. These findings may allow for a shortened and more streamlined assessment bundle to be developed, which could help to signpost clinicians toward those at the highest risk of not receiving adjuvant chemotherapy, allowing for early intervention with prehabilitation and post-operative rehabilitation, to improve function and prevent decline.

### Limitations

The results of this study need to be interpreted in light of its limitations. Primarily, the ‘Fast Recovery’ programme was only funded for a 12-month period, which restricted the number of patients that could be recruited. As such, comparisons between eras may have been limited by statistical power, meaning that only larger effect sizes would have been detectable and that some non-significant comparisons may represent false negatives. In addition, not all patients completed the full bundle of pre- and post-operative assessments, which further reduced the sample size for the analyses of these outcomes, and may have introduced selection bias. Finally, additional details relating to adjuvant chemotherapy, such as the timing relative to surgery, were not recorded, owing to difficulties in accessing the full patient notes for those treated in other centres. As such, it was not possible to assess the impact of the ‘Fast Recovery’ programme on these outcomes.

## Conclusions

Although the ‘Fast Recovery’ programme did not lead to a significant increase in adjuvant chemotherapy uptake, it was associated with significantly lower post-operative weight loss. Of the various scores included in the assessment bundle, those relating to physical performance appeared to be the best predictors of the receipt of adjuvant chemotherapy and may have utility in identifying patients who are more likely to require additional support with pre- and post-habilitation.

### Supplementary Information

Below is the link to the electronic supplementary material.

Supplementary file1 (DOCX 18 KB)

## Data Availability

All data generated or analysed during this study are included in this published article (and its supplementary information files).
