# Supplementary material for: Are physical performance and frailty assessments useful in targeting and improving access to adjuvant therapy in patients undergoing resection for pancreatic cancer?
Source: Langenbecks Arch Surg. 2023 Feb 14;408(1):88. doi: 10.1007/s00423-023-02828-1 (PMC9928938; doi:10.1007/s00423-023-02828-1)
Supplement: Supplementary file 1 — Supplementary file1 (DOCX 18 KB) [file 423_2023_2828_MOESM1_ESM.docx]

***Supplementary Table 1 – Subgroup analysis of CA19-9 in patients with jaundice***

|  | **Fast Recovery** | | | |  |
| --- | --- | --- | --- | --- | --- |
|  | ***No*** | | ***Yes*** | |  |
|  | N | *Median (IQR)* | *N* | *Median (IQR)* | **p-Value** |
| Pre-operative Biliary Drainage |  |  |  |  |  |
| *No* | 81 | 200 (58 - 588) | 31 | 443 (156 - 1506) | **0.013** |
| *Yes* | 216 | 173 (53 - 707) | 7 | 191 (41 - 626) | 0.861 |

*Data are reported as median (interquartile range) in U/ml, with p-value from Mann-Whitney U tests. Bold p-values are significant at p<0.05.*

**Supplementary Table 2 – *Comparison of complication rates between eras***

|  |  | **Fast Recovery** | |  |
| --- | --- | --- | --- | --- |
|  | **N** | ***No*** | ***Yes*** | **p-Value** |
| Any Complication | 453 | 163 (39.9%) | 16 (36.4%) | 0.746 |
| Any Clavien-Dindo Grade 3+ Complication | 453 | 57 (13.9%) | 6 (13.6%) | 1.000 |
| Comprehensive Complication Index | 453 | 12.4* | 10.9* | 0.770 |
| Complication Types | 453 |  |  |  |
| *Wound Infection* |  | 45 (11.0%) | 5 (11.4%) | 1.000 |
| *Post-operative Pancreatic Fistula*  *Biochemical leak*  *Grade B*  *Grade C* |  | 45 (11.0%)  18 (4.4%)  17 (4.2%)  10 (2.4%) | 4 (9.1%)  1 (2.3%)  2 (4.5%)  1 (2.3%) | 1.000  1.000  0.706  1.000 |
| *Intra-Abdominal Collection* |  | 29 (7.1%) | 2 (4.5%) | 0.756 |
| *Delayed Gastric Emptying* |  | 25 (6.1%) | 2 (4.5%) | 1.000 |
| *Gastrointestinal Bleed* |  | 21 (5.1%) | 3 (6.8%) | 0.719 |
| *Cardiac* |  | 21 (5.1%) | 2 (4.5%) | 1.000 |
| *Chest Infection* |  | 20 (4.9%) | 1 (2.3%) | 0.709 |
| *Renal* |  | 10 (2.4%) | 2 (4.5%) | 0.328 |
| *Acute Respiratory Distress Syndrome* |  | 11 (2.7%) | 0 (0.0%) | 0.611 |
| *Pseudoaneurysm (GDA)* |  | 3 (0.7%) | 2 (4.5%) | 0.076 |
| *Pulmonary Embolism/Deep Vein Thrombosis* |  | 4 (1.0%) | 0 (0.0%) | 1.000 |
| *Cerebrovascular Accident / Stroke* |  | 2 (0.5%) | 0 (0.0%) | 1.000 |
| *Dehiscence* |  | 1 (0.2%) | 0 (0.0%) | 1.000 |
| *Other Complication* |  | 41 (10.0%) | 7 (15.9%) | 0.298 |

*Data are reported as N (%), with p-values from Fisher’s exact test, or as median (IQR), with p-values from Mann-Whitney U tests, unless stated otherwise. Bold p-values are significant at p<0.05. *Reported as means, with p-value from Mann-Whitney U test. GDA=Gastroduodenal artery*
